# The influence of social media on young athletes’ mental health: a cross-sectional study

**DOI:** 10.3389/fpsyg.2026.1811861

**Published:** 2026-06-19

**Authors:** Camille Tooth, Jean-François Kaux, Philippe Tscholl, Alioune Touré, Frank Muller, Katy Seil

**Affiliations:** 1 ReFORM IOC Research Centre for Prevention of Injury and Protection of Athlete Health, Liège, Belgium; 2 Department of Physical Medicine, Rehabilitation and Sports Traumatology, SportS², FIFA Medical Centre of Excellence, FIMS Collaborative Centre of Sports Medicine, CHU de Liege, Liège, Belgium; 3 Luxembourg Institute of Research in Orthopedics Sports Medicine and Science (LIROMS), Luxembourg, Luxembourg; 4 Department of Physical Activity and Rehabilitation Sciences, Universite de Liege, Liège, Belgium; 5 Department of Orthopedic Surgery and Traumatology, Geneva University Hospitals, Geneva, Switzerland; 6 Service national de l’éducation inclusive au MENJE, Luxembourg, Luxembourg; 7 SportLycée, Luxembourg, Luxembourg; 8 Luxembourg Institute for High Performance in Sports (LIHPS), Luxembourg, Luxembourg

**Keywords:** athlete, health, online abuse, safeguarding, social media

## Abstract

**Introduction:**

Social media use has become highly prevalent among young athletes. While social media platforms can promote social connection and support, they may also contribute to stress, anxiety, sleep disturbances, body image concerns and online abuse. However, limited research has specifically explored these effects in athletic populations. This study aimed to assess the influence of social media on young athletes’ mental health and to examine whether age, sex, sport type, competitive level and platform used affected these perceptions.

**Methods:**

A cross-sectional online survey was completed by 912 athletes aged 12–24 years. The questionnaire explored social media exposure, perceived impact on wellbeing and performance, self-regulation strategies and experiences of online abuse. Associations between athlete characteristics and reported outcomes were analysed using multivariable logistic regression models.

**Results:**

Daily use of at least one platform was reported by 93.4% of athletes, and 63.6% indicated that social media interfered with training or recovery. Perceived effects were ambivalent: 57.1% reported receiving encouragement or sport-related support online, whereas 47.7% reported negative effects on sleep quality and 25.4% reported stress or anxiety related to social media use. Pressure to maintain an online image was reported by 29.2% of athletes and was more frequent among females and older athletes. Greater exposure time was associated with stronger negative effects on sleep, mood, self-image and feelings of isolation. Online abuse was reported by 10.4% of athletes, although only a minority sought support.

**Discussion:**

Social media plays a dual role in young athletes’ lives, providing opportunities for connection and support while also exposing them to psychological vulnerabilities and digital pressures. These findings highlight the importance of targeted education, safeguarding strategies and monitoring of digital habits, as usage volume appears to be a key determinant of impact.

## 1 Introduction

The development and the increase of social media use has implications for the mental health of young people. Recent statistics show that 95% of individuals aged 13 to 27 engage with platforms like YouTube, TikTok, Instagram and Snapchat, with 35% reporting near-constant usage (Datareportal, 2023; Putukian et al., 2024). This trend has raised important concern among international health authorities, including the U.S. Surgeon General and the American Psychological Association, both of which have identified social media as a critical factor in the current youth mental health crisis (American Psychological Association, 2023; HHS, 2023; Putukian et al., 2024).

For young athletes, who already operate under heightened pressure due to competition, visibility and identity research around performance, the impact of digital exposure is particularly complex. Social media plays a dual role: on one hand, it can increase social connections, peer support and provide opportunities for self-expression; on the other hand, it can induce increased anxiety, social comparison and pressure to maintain a certain image, especially when usage is frequent and unregulated (whether at the level of the athlete, the entourage or the platform itself) (Alonzo et al., 2021; Mossberg and Möllborg, 2023; Kavanagh and Mountjoy, 2024).

Sleep disturbances are among the most consistently reported consequences of excessive screen time. Evening and nighttime usage disrupts rest and recovery cycles, which are extremely important for both psychological wellbeing and athletic performance (Alonzo et al., 2021). For adolescent athletes, this can create a vicious cycle in which poor sleep exacerbates emotional vulnerability, leading to further online dependence and distress (Mossberg and Möllborg, 2023).

Online abuse further increases this vulnerability. It is not limited to isolated events but tends to be repetitive, public and difficult to escape. Online abuse^1^ often overlaps with offline psychological or physical harm, reinforcing feelings of isolation and helplessness (Kavanagh and Mountjoy, 2024; Tuakli-Wosornu et al., 2024). Female, disabled athletes or athletes from minorities are especially at risk, as they more frequently report sexualised or racialised harassment (Vertommen et al., 2016; Tuakli-Wosornu et al., 2024). These patterns highlight the need for context-specific digital safeguarding strategies that address online abuse.

In response to these developments, the International Olympic Committee has recently updated its definition of safeguarding to explicitly include digital spaces and online behaviours (Tuakli-Wosornu et al., 2024). Despite the growing body of literature on social media and youth mental health, several gaps remain. Research focusing specifically on the mental health effects of social media among young or elite athletes is still limited. Besides, few studies explored how factors like gender, level of sport, type of sport or social media platform may shape digital experiences and psychological (positive and negative) outcomes (Kavanagh and Mountjoy, 2024). In addition, few studies have simultaneously explored multiple dimensions of social media use, including exposure, perceived impact and behaviours such as self-regulation and responses to online abuse.

Therefore, this study aims to address these gaps by examining the effects of social media use, on young athletes’ mental health. This project seeks to better understand the impact on sleep, emotional wellbeing, performance-related stress, and body image, in order to better inform targeted prevention and support strategies among this population.

## 2 Methods

### 2.1 Participant recruitment

Young athletes aged between 12 and 24 years were recruited for the study. This age group was chosen because 24 years is considered as the end of adolescence (Sawyer et al., 2018). They were recruited through the scientific partners, the ReFORM network as well as through Olympism365 (representing local organisations involved in implementing IOC-supported sport and development initiatives), between February and May 2025. The questionnaire was disseminated in three languages (French, English, and German) via institutional email campaigns, social media posts and through affiliated communication channels to ensure broad reach. To be eligible, athletes had to be actively training or competing within a club or a federation and able to complete the questionnaire independently.

Before accessing the survey, participants were presented with a clear and accessible information notice outlining the purpose of the study, the voluntary nature of participation (no direct pressure from coaches or authority), and data confidentiality. Informed consent was obtained online by validating participation through the survey platform. The study was reviewed and approved by the Medical Ethics Committee of the University of Liège (B7072024000076) and complied with all applicable ethical standards. The questionnaire was fully anonymous. No names or identifying information were collected and all data were stored on a secure server compliant with GDPR standards. To ensure methodological rigour, STrenghtening the Reporting OBservational Studies in Epidemiology (STROBE) guidelines as well as CHAMP checklist (Checklist for statistical Assessment of Medical Papers) were followed (Mansournia et al., 2021).

### 2.2 Development of the questionnaire

The questionnaire was developed by a multidisciplinary team composed of researchers and clinicians in sports medicine, psychology and psychiatry. Its purpose was to explore the relationship between social media use and the mental health of young athletes.

The development process followed several key steps. First, a literature review was conducted to identify the most relevant psychological and behavioural dimensions associated with social media use in adolescent and athlete populations. An initial draft of the questionnaire was then created and reviewed by the research team. The wording and structure were revised to ensure age-appropriate phrasing. A secure online survey platform was selected to host the questionnaire (Microsoft Forms), allowing for adaptive questioning, multilingual access, and optimized formatting.

The questionnaire was pilot tested with a sample of 13 adolescent athletes to assess clarity, flow, and response time. Feedback from this pilot phase was used to finalize the structure before broader dissemination.

### 2.3 Content of the questionnaire

As no validated instrument was available to assess all dimensions explored in this study, the questionnaire was developed using items informed by existing literature and previous survey-based studies on social media use and its perceived effects (Thygesen et al., 2021). The final version of the questionnaire included 35 questions and was divided into six main sections. The structure was as follows:

Part 1—General and sport-related information: this section gathered basic demographic and contextual information including age, gender, sport and type of sport (individual vs. team sport, based on standard definitions), level of competition (using McKay et al. classification) (McKay et al., 2021).
Part 2—Social media use: athletes were asked to indicate the platforms they used most frequently (e.g., Instagram, TikTok, Snapchat, YouTube etc.), the estimated time spent online per day, typical moments of use (e.g., before bedtime, after training), main motivations for their use (e.g., entertainment, communication, sport-related content, peer validation) etc.
Part 3—Perceived impact of social media: this section assessed athletes’ perceptions of how social media affected several domains of their mental and physical wellbeing, including self-confidence, sleep quality, mood, eating habits etc.
Part 4—Perceived sport-specific impact of social media: a specific section was dedicated to examining how athletes perceived the interaction between digital media and their sport. It included questions on image rights and issues around social media exposure, perceived performance pressure etc.
Part 5—Hyperconnectivity (means being constantly connected trough digital systems) and self-regulation strategies: these aspects were assessed based on athletes’ self-reported behaviours and perceptions, including time spent on social media, its interference with training or recovery, and attempts to regulate their usage (through apps, screen time restrictions etc.).
Part 6—Online abuse: the final section focused on exposure to online abuse. Athletes were asked whether they had experienced online abuse at least once in their lifetime (in relationship with sport or not). Follow-up questions examined the emotional consequences of such experiences (e.g., anxiety, loss of sleep, reduced self-esteem), and whether any support was sought. The section also assessed knowledge of reporting procedures and perceived access to help.

Most constructs were assessed using dichotomous, categorical or Likert-type response scales. For example, perceived mental health impact was explored using a 5-point Likert scale ranging from “Not at all” to “Extremely” in response to the question: “On a scale of 1 to 5, how much do you think social media usage influences your mental health?”. Pressure related to online image was assessed through the item: “Have you ever felt pressure to maintain a certain image of yourself on social media?” while social comparison tendencies were explored using the question: “Do you compare yourself to other athletes/individuals you see on social media?*” with response options* “Yes, often,” “Yes, sometimes,” and “No, never.” Perceived interference with sport participation and recovery was explored through questions such as: “Do you think the time spent on social media encroaches on the time you could dedicate to training, resting, or leisure activities?”. Online abuse was assessed using the item: “Have you ever been a victim of online abuse (negative comments, hateful messages, online defamation, etc.)?”, and its perceived consequences on domains such as self-confidence, sleep quality, mood, performance, desire to train and eating habits were rated using a severity scale ranging from “No impact” to “Very severe impact”. The full questionnaire is available from the corresponding author upon request.

### 2.4 Data processing and statistical analysis

Analyses were performed using SAS software (version 9.4). Categorical variables were described using frequency tables (number and percent) and quantitative variables were described using means and standard deviation (± SD) as well as extreme values. The categorical variables were compared using the Chi-square test.

To explore other determinants of the impact of social media use, the following factors were examined: sex, age group, type of sport, social media platforms, requirement to relinquish image rights, involvement in sports commonly associated with betting and level of practice. Univariate logistic regression models were first computed to test the independent effect of each factor. Variables with a *p*-value below 0.10 in univariate analysis were then entered into multivariable logistic regression models. Regarding the platforms used (e.g., Snapchat, TikTok, Instagram…), six variables were generated representing the use (or not) of each platform (0 = no, 1 = yes). Because these variables reflected complementary aspects of social media exposure and athletes frequently used several platforms simultaneously, all platform variables were entered jointly into the multivariable models whenever at least one platform variable reached the predefined threshold in univariate analyses. Results were expressed as odds ratios (OR) with their 95% confidence intervals and corresponding *p*-values. No adjustment for multiple testing was applied. All the results were considered statistically significant at the 5% level (*p* < 0.05).

## 3 Results

In total, 968 young athletes responded to the survey, of whom 912 met the inclusion criteria and completed the questionnaire in full (385 females; 522 males; mean age of 16.5 ± 3.1 years). Among them, 38.7% practiced an individual sport and 61.3% practiced a team sport. The main sports represented were rugby (32.7%), followed by athletics (11.5%), soccer (10.5%) and basketball (9.2%). Among the sample, 26% are considered as “amateur athletes”, 26.2% as “competitive athletes”, 33.2% as “high-level athletes”, 13.2% as “elite athletes” and 1.3% as “World class athletes”. Given the low number of athletes included in the “World Class” category and in order to increase statistical power, their results were analysed with those from elite athletes. All demographic data are presented in detail in Table 1.

**Table 1:** Demographic data (*N* = 912).

Variables	N (%)
Sex	
Female	385 (42.2)
Male	522 (57.2)
Non-binary	2 (0.2)
Prefer not to say	3 (0.3)
Age (Years), Mean ± SD	16.5 ± 3.1
Country	
Luxembourg	425 (46.6)
France	338 (37.1)
Belgium	82 (9.0)
Switzerland	28 (3.1)
Canada	19 (2.1)
Italy	4 (0.4)
Ireland	2 (0.2)
Other countries	14 (1.5)
Main sport	
Individual sport	353 (38.7)
Team sport	559 (61.3)
Level of practice	
World Class	12 (1.3)
Elite	121 (13.3)
High-level	303 (33.2)
Competitive	239 (26.2)
Amateur	237 (26.0)

To facilitate reading, results were presented in four steps: social media use (1), self-reported impact (2), self-regulation (3) and online abuse (4). Descriptive findings were reported using percentages. Group comparisons were performed using Chi-square tests. Finally, multivariable analyses were presented using odds ratios (OR) with 95% confidence intervals.

### 3.1 Social media use

**Figure 1:**
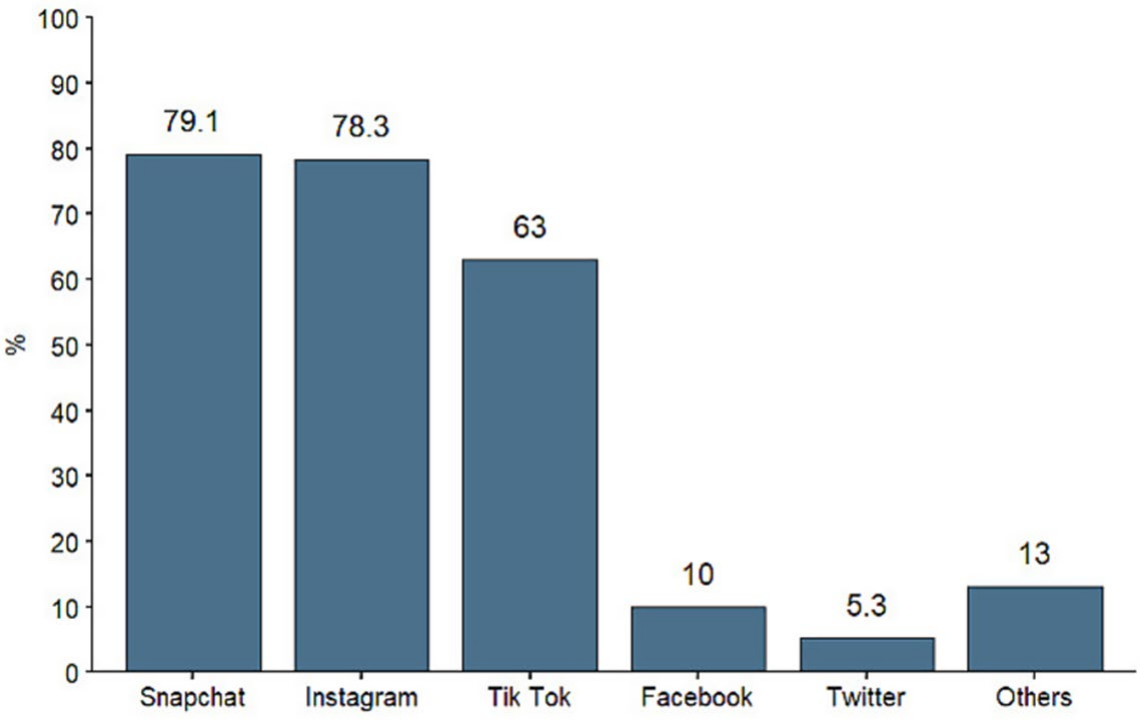
Social media use (*N* = 912 young athletes).

Overall, 93.4% of the young athletes report using at least one social media platform. The most popular are Snapchat (79.1%), Instagram (78.3%), and TikTok (63.0%), while Facebook and Twitter were used by only 10.0 and 5.3%, respectively. In terms of exposure, two-thirds of athletes (67.7%) spend more than 2 h per day on social media, and 81.8% also used other types of media (films, video games etc.) (Figure 1).

Social media are primarily used to communicate with friends or family (79.3%) and for leisure or distraction (70.8%). More than half of the athletes (55.2%) follow sports news through these platforms and 43.6% use them to share or view content related to their sport. Notably, 59.6% acknowledge that they compared themselves with others at least occasionally.

Social media use does not differ significantly according to sex or age (*p* = 0.90 and *p* = 0.24, respectively), but those variables are both significantly associated with the volume of use reported by the athletes (Table 2). Among girls, 71.4% report spending more than 2 h per day on social media compared with 64.9% of boys (*p* = 0.039). The likelihood of using social media for more than 2 h daily also increases with age (*p* < 0.0001).

**Table 2:** Factors influencing social media use (*N* = 912).

Variables	Use of at least one social media network		Volume social media > 2 h/day	
	n/N (%)	*p-*value	n/N (%)	*p*-value
Sex (Ref = Male)		0.90		0.039*
Female	360/385 (93.5)		275/385 (71.4)	
Male	487/522 (93.3)		339/522 (64.9)	
Age (Years), OR (95% CI)	0.95 (0.88–1.03)	0.24	1.2 (1.1–1.3)	<0.0001*
Sport		0.0012*		0.0009
Individual	318/353 (90.1)		216/353 (61.2)	
Collective	534/559 (95.5)		401/559 (71.7)	
Main sport				–
Rugby	281/298 (94.3)		211/298 (70.8)	
Athletics	93/105 (88.6)		67/105 (63.8)	
Soccer	93/98 (94.9)		67/98 (68.4)	
Basketball	83/84 (98.8)		59/84 (70.2)	
Handball	53/54 (98.1)		43/54 (79.6)	
Combat sport	41/47 (87.2)		26/47 (55.3)	
Racket sports	41/44 (93.2)		29/44 (65.9)	
Shooting	34/36 (94.4)		28/36 (77.8)	
Dance	19/23 (82.6)		11/23 (47.8)	
Triathlon	19/21 (90.5)		10/21 (47.6)	
Gymnastics	19/20 (95.0)		15/20 (75.0)	
Volleyball	17/17 (100.0)		14/17 (82.3)	
Swimming	11/12 (91.7)		8/12 (66.7)	
Cycling	10/10 (100.0)		5/10 (50.0)	
Ski	7/8 (87.5)		3/8 (37.5)	
Horse riding	6/6 (100.0)		4/6 (66.7)	
Fencing	5/5 (100.0)		2/5 (40.0)	
Level of practice		0.30		0.0012*
Amateur	227/237 (95.8)		177/237 (74.7)	
Competitive	224/239 (93.7)		165/239 (69.0)	
High-level	279/303 (92.1)		180/303 (59.4)	
Elite or World Class	122/133 (91.7)		95/133 (71.4)	

For categorical variables results are expressed as number and percent and *p*-value Chi-square test, for continuous variable logistic regression models were applied and results are odd ratio (OR), 95% confidence interval of OR and *p*-value.

Team sport athletes are more active online than those in individual sports. While 90.1% of athletes from individual sports use social media, this proportion raises to 95.5% among team sport athletes (*p* = 0.0012). Similarly, the proportion of athletes spending more than 2 h per day on social media is significantly higher in team sports (*p* = 0.0009). Across the most represented disciplines (≥ 5 respondents), the percentage of social media users (at least 1 platform) ranges from 82.6% (dance) to 100% (volleyball, cycling, horse riding, fencing). The proportion of athletes spending more than 2 h daily on social media varies widely between sports, from 37.5% among skiers to 82.3% among volleyball players.

No significant association has been found between competitive level and the likelihood of using social media (*p* = 0.21). However, athletes at a competitive or high-level of practice are less likely to report using social media for more than 3 h per day compared with amateurs (63.7% vs. 74.7%, *p* = 0.0062).

### 3.2 Self-reported impact of social media

Table 3 summarises athletes’ perceptions of how social media use affects their health, well-being and sport performance. Overall, 64.1% of respondents describe its influence on health as both positive and negative, illustrating the ambivalence of its perceived effects. While 57.1% reported having received encouragement or support via social media that helped them in their sporting career, many also recognise negative impacts, particularly concerning sleep. Nearly half of the athletes (47.7%) feels that social media has a negative impact on their sleep quality, and almost two-thirds (63.6%) report that time spent online interferes with their training or rest.

**Table 3:** Impact of social media use (*N* = 912).

Variables	N (%)
Influence on health	
Not at all	125 (13.7)
A little	323 (35.4)
Moderately	296 (32.5)
Significantly	141 (15.5)
Extremely	27 (3.0)
Negative	107 (11.7)
Positive	220 (24.1)
Positive and negative	585 (64.1)
Impact on stress and anxiety	232 (25.4)
Impact on self confidence	
No impact	428 (46.9)
Positive impact	122 (13.4)
Negative impact	146 (16.0)
Do not know	216 (23.7)
Impact on quality of sleep	
No impact	331 (36.3)
Positive impact	25 (2.7)
Negative impact	435 (47.7)
Do not know	121 (13.3)
Impact on mood	
No impact	489 (53.6)
Positive impact	121 (13.3)
Negative impact	143 (15.7)
Do not know	159 (17.4)
Impact on desire to train	
No impact	419 (45.9)
Positive impact	376 (41.2)
Negative impact	50 (5.5)
Do not know	67 (7.3)
Impact on performance	
No impact	497 (54.5)
Positive impact	201 (22.0)
Negative impact	48 (5.3)
Do not know	166 (18.2)
Impact on self-image	
No impact	481 (52.7)
Positive impact	106 (11.6)
Negative impact	154 (16.9)
Do not know	171 (18.8)
Impact on eating habits	
No impact	539 (59.1)
Positive impact	189 (20.7)
Negative impact	97 (10.6)
Do not know	87 (9.5)
Impact on ability to focus on training and competitions	
Very negative	9 (1.0)
Rather negative	81 (8.9)
Neutral	657 (72.0)
Rather positive	125 (13.7)
Very positive	40 (4.4)
Pressure to maintain image	266 (29.2)
Relinquish image rights sponsors	199 (21.8)
Feeling on isolation vs connection	
No effect	536 (58.8)
More connected	294 (32.2)
More isolated	70 (7.7)
Both	8 (0.9)
Other or do not know	4 (0.4)
Received support must be bigger as other variables	521 (57.1)

Moreover, looking at the negative effects, an important proportion of athletes (29.2%) feels pressure to maintain a certain image online, and one quarter (25.4%) associates social media with stress or anxiety. Regarding self-confidence, most respondents (46.9%) perceive no direct impact, while 13.4% described a positive effect and 16.0% a negative one. The reported impact on mood is mixed: 15.7% identified a negative effect and 13.3% a positive one.

When asked about sport-specific consequences, 41.2% believe that social media has a positive influence on their desire to train, whereas 5.5% report a negative one. Similarly, 22.0% perceived a positive impact on performance, while only 5.3% mention a negative effect.

Concerning social relationships, the majority (58.8%) feel that social media had no particular effect on their sense of connection, although 32.2% report feeling more connected and 7.7% being more isolated.

Finally, 21.8% of athletes indicate having relinquished certain image rights for sponsors or promotional purposes.

#### 3.2.1 Influence of the level of practice

**Figure 2:**
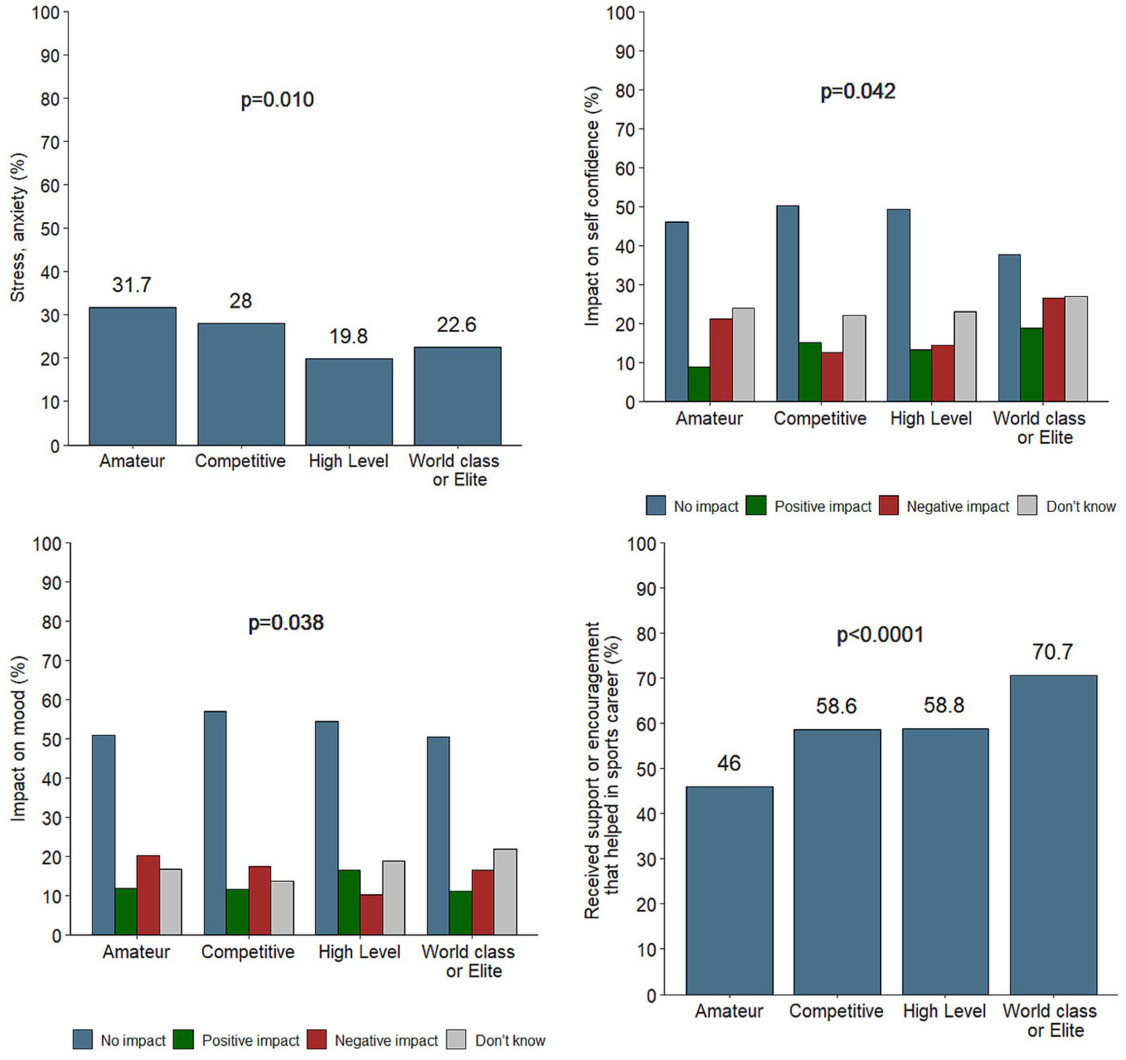
Impact of social media use in function of the level of practice (*N* = 912).

The impact of social media use differs significantly across competitive levels (Supplementary material 2 and Figure 2). Negative effects on stress and anxiety are more frequently reported among amateur (31.7%) and competitive athletes (28.0%) than among high-level (19.8%) and world-class/elite athletes (22.6%) (*p* = 0.010). Perceived effects on self-confidence also significantly vary across levels of practice (*p* = 0.042), with world-class/elite athletes more often reporting both positive and negative impacts. Mood is similarly influenced by the level of practice (*p* = 0.038): high-level athletes report more positive effects and fewer negative effects than other groups. Differences are also observed regarding image rights and online visibility, which are more frequently maintained for sponsorship or professional purposes among world-class/elite athletes (32.3%) compared with amateurs (23.6%), competitive athletes (15.9%) and high-level athletes (20.5%) (*p* = 0.0025). Then, the proportion of athletes who received support or encouragement through social media that helped their career increases with competitive level (*p* < 0.0001).

#### 3.2.2 Influence of number of hours spent on social media

**Figure 3:**
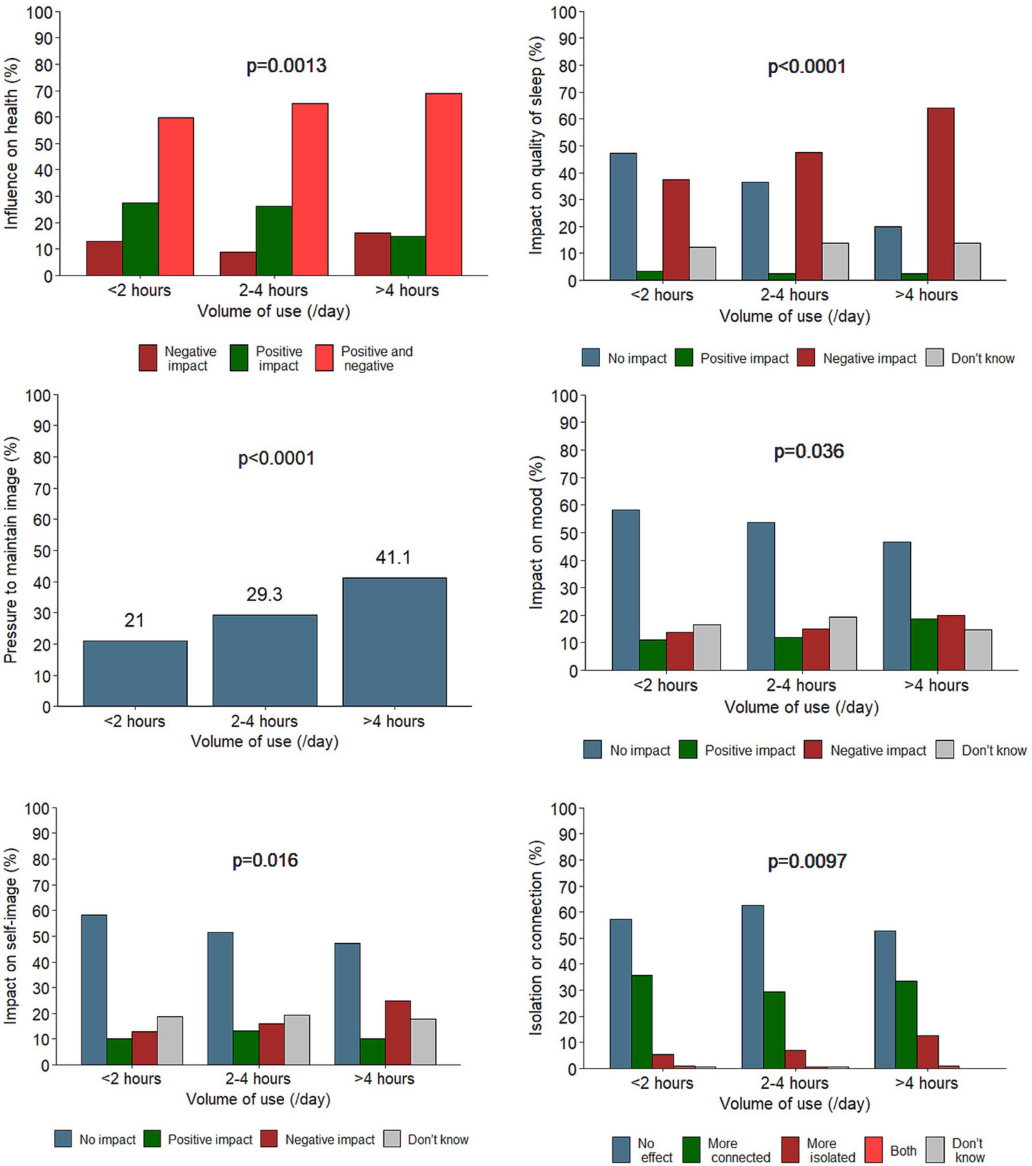
Impact of social media use according to the athlete, in function of the time spent online (*N* = 912).

The overall perceived impact on health increases with higher exposure (*p* = 0.0056), as does the pressure to maintain an online image (*p* < 0.0001) (Figure 3). Negative effects on sleep also increase with the number of hours spent online (*p* < 0.0001). Moreover, greater exposure is associated with more impact on mood (*p* = 0.036), desire to train (*p* = 0.014) and self-image (*p* = 0.016), including stronger negative components. Feelings of isolation are also significantly more prevalent at higher exposure levels (*p* = 0.0097). Simultaneously, relinquishing image rights or having to publish content due to sponsorship is more frequent among heavy users (*p* = 0.031), as are positive and negative effects on motivation and athletic performance (*p* = 0.0065).

#### 3.2.3 Other factors influencing social media impact

Multivariable analyses reveals that several characteristics independently influence how athletes perceive the impact of social media use on their well-being and performance (Supplementary material 3). The first factor is the sex of the athletes. Female athletes are significantly more likely than males to report pressure to maintain an image (adjusted OR = 1.7; 95% CI 1.2–2.2; *p* = 0.0013), stress/anxiety related to social media (aOR = 1.7; 95% CI 1.2–2.3; *p* = 0.0012), as well as negative effects on self-confidence (aOR = 2.2; 95% CI 1.5–3.0; *p* < 0.0001). Female athletes are also more frequently affected in terms of self-image (aOR = 2.3; 95% CI 1.7–3.1; *p* < 0.0001) and eating behaviours (aOR = 1.6; 95% CI 1.2–2.1; *p* = 0.0027).

Age is another significant determinant. Young adults (>18 years) report greater pressure to maintain image (aOR = 1.6; 95% CI 1.2–2.3; *p* = 0.0037) and poorer sleep quality (aOR = 1.8; 95% CI 1.3–2.6; *p* = 0.0004).

Regarding social media platforms, Instagram use correlates with stronger self-perceived impacts on self-confidence (aOR = 1.8; 95% CI 1.1–3.1; *p* = 0.023), desire to train (aOR = 1.7; 95% CI 1.1–2.6; *p* = 0.024) and motivation (aOR = 1.7; 95% CI 1.1–2.5; *p* = 0.012). TikTok, in contrast, is associated with sleep disturbances (aOR = 1.5; 95% CI 1.1–2.2; *p* = 0.017). As to Twitter, the network is linked to both negative effects on sleep (aOR = 2.6; 95% CI 1.1–5.7; *p* = 0.023) and decreased self-confidence (aOR = 2.3; 95% CI 1.1–4.6; *p* = 0.019).

Then, athletes who had relinquished image rights to sponsors or organisations are significantly more exposed to image-related pressure (aOR = 2.3; 95% CI 1.7–3.3; *p* < 0.0001) and report stronger impacts on self-confidence (aOR = 2.2; 95% CI 1.5–3.3; *p* < 0.0001), self-image (aOR = 1.7; 95% CI 1.2–2.6; *p* = 0.0025) and feelings of isolation (aOR = 2.3; 95% CI 1.4–3.8; *p* = 0.0009).

Finally, considering type of sport, those involved in team sports appear less affected overall. Team environments are associated with lower odds of negative impacts on self-confidence (aOR = 0.65; 95% CI 0.46–0.92; *p* = 0.014) and motivation (aOR = 0.62; 95% CI 0.47–0.82; p = 0.0009). Conversely, athletes in sports linked to betting activities (soccer, combat sports, racquet sports or horse riding) shows higher susceptibility to stress and anxiety (aOR = 1.5; 95% CI 1.1–2.0; *p* = 0.0085).

### 3.3 Self-regulation

Overall, 63.6% reports that time spent online interfered with their training or recovery and 59.6% has felt the need to take a break or limit their use. Yet only 42.1% has actually implemented concrete strategies.

The proportion of athletes who perceives social media as encroaching on training increases with exposure time, from 55.2% among those using it less than 2 h per day to 76.7% among those exceeding 4 h (*p* < 0.0001). Conversely, the likelihood of implementing self-regulation strategies decreases with greater exposure (54.2, 39.8, and 28.9% respectively; *p* < 0.0001).

Athletes in individual sports are more likely to take proactive measures than those in team sports (47.9% vs. 38.5%; *p* = 0.005), while high-level athletes are the least likely to express the need to limit their use (*p* = 0.049). Instagram use is associated with a higher risk of interference with training or rest (aOR = 1.7; 95% CI 1.2–2.6; *p* = 0.0074), whereas TikTok users are less likely to report having implemented limiting strategies (aOR = 0.46; 95% CI 0.33–0.64; *p* < 0.0001).

### 3.4 Online abuse

Online abuse was reported by 10.4% of the athletes surveyed. Among victims (Supplementary material 4), more than half reports moderate to severe effects on mood (37.9%), sleep quality (44.3%) and self-confidence (45.3%), while one third experienced significant consequences on self-image and training motivation. Although only a minority describes the impact as “very severe,” these effects are often cumulative across domains. The prevalence of online abuse differs significantly by competitive level (*p* < 0.0001): non-sport-related online abuse is more common among amateurs, whereas sport-related online abuse is more frequently reported by world-class or elite athletes. Among those affected, 35.8% sought help, mostly from friends or family (64.7%), while only a small proportion reported to health professionals (20.6%) or coaches (5.9%).

## 4 Discussion

The findings of this study suggest that the influence of social media on young athletes is neither only negative nor positive but instead characterised by a combination of beneficial and detrimental effects, which have a significant impact on their performance. A large proportion of respondents reported that social media had both positive and negative influences on their health, which echoes previous literature describing social media as an ambivalent environment for adolescents (Uhls et al., 2017; O’Reilly, 2020). While many athletes identified encouragement, access to sport-related information and increased motivation through their online interactions, the same platforms were also associated with increased stress, self-comparison, sleep disturbances as well as pressure to maintain a certain image online. These results are in line with recent international recommendations emphasising that social media carries both opportunities and risks for youth mental health (American Psychological Association, 2023; HHS, 2023; Scully et al., 2023).

Besides, the influence of social media is visible across all levels of practice, although with different patterns. Amateur and competitive athletes reported higher levels of stress and anxiety linked to social media compared to high-level or elite athletes. This trend may reflect differences in media training, maturity or access to support structures. Adolescents with less experience in managing public visibility or sports-related pressure may be more strongly impacted by social comparisons than others, as highlighted in previous studies examining body image and appearance-related content among youth (Rumbold et al., 2012; Prasad et al., 2023; Scully et al., 2023). At the same time, in the current study, athletes with greater visibility (in individual sports or in sports frequently associated with betting activities), reported more impact of social media on variables such as mood or desire to train than the other sports practitioners. This observation aligns with recent studies and newspaper showing a growing proportion of online hostility in sports such as tennis, tennis table, soccer, combat sports or horse riding, which may significantly impact the athlete, due to external financial interests (Kavanagh and Mountjoy, 2024). According to the results of this study, age may also play a role, with older athletes reporting stronger pressure to maintain an online image and greater sensitivity to comparison mechanisms than younger athletes.

Beyond mental wellbeing, the present study also highlights that social media use may affect several determinants of performance. Nearly half of the athletes reported negative effects on sleep and the prevalence of those negative effects increased with the time spent on social media. These findings are consistent with research indicating that evening or excessive use disrupts sleep quality and recovery processes (Woods and Scott, 2016; Alonzo et al., 2021). Considering that sleep is a key element of physiological restoration and injury prevention, the association between digital habits and sleep patterns has direct implications for athletic performance (Charest and Grandner, 2020). In addition, negative effects of social media were observed on self-confidence and self-image. Female athletes reported these impacts more frequently, which is coherent with literature describing heightened vulnerability to appearance-related comparisons and online pressures among adolescent girls (Scully et al., 2023). Changes in eating behaviours linked to social media are also worrying, as misinformation and restrictive trends circulating online can influence nutritional practices in adolescents (Kucharczuk et al., 2022).

Importantly, 63.6% of athletes felt that social media interfered with their training or recovery, yet less than half had implemented strategies to limit their use. This discrepancy reflects findings in adolescent populations more broadly, where problematic use is recognised but behavioural change remains limited (Mossberg and Möllborg, 2023). In the context of sport, this lack of regulation may be reinforced by social expectations, fear of losing visibility or sponsorship requirements (Grohs and Reisinger, 2005). The present results therefore tend to underline that social media cannot be considered solely a mental health issue; it must also be approached as a factor influencing performance management, with potential repercussions for motivation, desire to train, training availability and recovery.

Online abuse constitutes another central dimension of these findings. Although 10.4% of athletes report having experienced online harassment, the consequences are substantial for those affected, with clear impacts on mood, sleep, motivation and self-image. These observations are also in line with recent international reports and consensus (United Against Online Abuse, n.d.; Tuakli-Wosornu et al., 2024) showing that online abuse is increasingly prevalent and often cumulative in its effects. The fact that elite athletes more frequently report sport-related online abuse is consistent with monitoring data from major competitions, where athletes with greater visibility are more likely to be targeted (Sloan et al., 2025). Despite the severity of the consequences, only a minority of victims are seeking help, and when they did, support primarily comes from family or friends. Very few turn to coaches, health professionals or safeguarding officers. This limited use of formal support systems has been reported in previous studies on digital behaviour in adolescents (Barry et al., 2017) and suggests that sport environments may not yet provide clear or accessible pathways for reporting or managing online harassment.

### 4.1 Practical implications

Taken together, these results highlight the need for structured educational initiatives aimed at athletes, parents, coaches and sport organisations. Digital literacy, recognition of risks, management of online visibility and early intervention strategies should be integrated into existing mental health and safeguarding frameworks. Recent consensus statements stress that safeguarding must now include digital spaces, given the rapid expansion of online abuse and the central role of social media in athletes’ daily lives (Kavanagh and Mountjoy, 2024; Putukian et al., 2024; Tuakli-Wosornu et al., 2024). The present findings provide support for these recommendations and emphasise the necessity of equipping athletes and their entourage with practical tools to manage a constantly evolving digital environment. As different effects are observed across age groups, sex, sport types (individual vs. team sport) and platforms, this further underscore the need for tailored preventive strategies that account for these specificities. Indeed, Instagram appears to have a stronger influence on self-image, as athletes are more frequently exposed to appearance-focused content through stories, whereas TikTok is more closely associated with addictive use, with its algorithms continuously generating new videos that encourage prolonged scrolling. Those factors have to be considered in a preventive and educational approach. Besides, since the volume of exposure is clearly associated with several negative outcomes, the objective should not be to ban social media from young athletes’ life but to recognise that the amount of time spent on these platforms is determinant for both mental wellbeing and performance and that monitoring the volume may therefore be relevant in future prevention strategies.

These results also invite reflection on the role of parents, coaches and the broader entourage in shaping athletes’ digital habits and responses to online pressures. Parents are often the first line of support, yet they may lack awareness of platform mechanisms or underestimate the impact of online content on motivation, sleep and self-image. Strengthening parental digital literacy, encouraging open discussions about online experiences and providing families with practical tools (monitoring routines, device-free periods, co-constructed rules) may also help adolescent athletes to decrease the negative impacts associated with social media. Similarly, coaches and support staff also have an important role in this domain. Future interventions should not only focus on athletes but should also include their entourage, ensuring that young people are supported by adults who understand the digital ecosystem and can help them navigate it safely and constructively.

That said, focusing only on education and behaviour change among athletes and their entourage may be too limited. It can also unintentionally place most of the responsibility on those who are exposed to these environments. Social media platforms themselves shape how content is shared, seen and amplified (Metzler and Garcia, 2024), and therefore have a role to play. The way algorithms work, how content is moderated, and how reporting systems are designed all influence the spread of harmful content. Strengthening reporting systems and implementing measures targeting users who engage in abusive behaviours are essential to create safer digital environments (Tuakli-Wosornu et al., 2024).

Finally, although the study did not directly assess the impact of artificial intelligence, emerging developments in content generation, algorithmic amplification and automated harassment mechanisms may further influence athletes’ online experience. Future research should also consider these aspects, as AI-driven systems increasingly influence the phenomenon and pressures to which young athletes are exposed.

### 4.2 Limitations

Several limitations must be acknowledged when interpreting the findings of this study. Firstly, all data were self-reported, which introduces risks of social desirability bias and subjective interpretation of questions. However, understanding perceived impact is important, as it reflects athletes’ level of awareness and interpretation of their own digital behaviours. Future studies should aim to complement these findings with more objective measures of social media use and its effects, in order to better understand the relationship between perceived and real impact. The reliance on adolescents’ and young adults’ perceptions may also lead to inaccuracies in estimating time spent online, sleep disturbances or the emotional impact of social media (Boyle et al., 2022). Secondly, although the sample size is substantial, the geographical distribution of athletes is uneven. Most respondents were from Europe and North America, while athletes from other regions of the World were underrepresented. This limits the generalisability of the results, especially given that access to digital tools, cultural norms, online behaviours and safeguarding structures vary greatly across countries. Thirdly, sport representation was heterogeneous, with some disciplines strongly represented (e.g., rugby) and others represented by small subsamples, potentially reducing statistical power to detect sport-specific patterns.

## 5 Conclusion

This study examined how social media affect the mental health of young athletes. Overall, the results suggest that social media plays a mixed role: it can be useful and supportive, but it can also have significant and negative effects on both mental health and performance. Many athletes highlighted positive aspects such as encouragement, access to sport-related information or increased motivation. At the same time, an important number reported sleep problems, more anxiety, frequent social comparisons and a sense of pressure linked to their online image. These observations suggest that social media use cannot be considered simply “good” or “bad,” but rather reflects a set of interacting factors, including the type of platform used, the athlete’s age, level of practice and the specific demands of their sport.

The study also suggest that digital behaviours influence areas that are directly related to performance. Sleep, concentration, eating habits and motivation were all affected to varying degrees and higher exposure was often associated with reduced recovery or less available training time. Then, the study highlighted that online abuse had clear consequences for those who experienced it, particularly on mood, self-confidence and motivation. Yet only a small number of athletes sought help, and most turned to family or friends rather than to coaches or health professionals or safeguarding officers.

Together, these findings support the importance of developing educational and preventive measures for athletes and their entourage. Improving digital literacy, raising awareness of risks and providing accessible pathways for support should form part of existing safeguarding and mental health strategies in sport. As digital tools continue to evolve, especially with the growing influence of AI, future studies will need to consider how these changes further impact young athletes’ well-being and performance.

## Data Availability

The raw data supporting the conclusions of this article will be made available on request by the authors, without undue reservation.

## Footnotes

1 Online abuse refers to the use of internet-connected technologies (such as smartphones, computers, and social media plateforms) to harm, exploit, intimidate, or threaten individual individuals or groups. These behaviours may be over or subtle, isolated or sustained, and can cause psychological, emotional, physical, sexual, reputationl, or economic harm. https://www.sportanddev.org/sites/default/files/2026-02/SSI_Paper_Online_Abuse_of_Athletes_2026-1.pdf.
