# Supplementary material for: The influence of social media on young athletes’ mental health: a cross-sectional study
Source: Front Psychol. 2026 Jun 19;17:1811861. doi: 10.3389/fpsyg.2026.1811861 (PMC13329723; doi:10.3389/fpsyg.2026.1811861)
Supplement: Supplementary file 1 [file Data_Sheet_1.docx]

**Supplementary material**

**Supplementary Material 1 - Main sports practiced by participants**

|  | N (%) |
| --- | --- |
| Rugby | 298 (32.7) |
| Athletics | 105 (11.5) |
| Football | 98 (10.7) |
| Basketball | 84 (9.2) |
| Handball | 54 (5.9) |
| Combat sport | 47 (5.2) |
| Racket sports | 44 (4.8) |
| Shooting | 36 (3.9) |
| Dance | 23 (2.5) |
| Triathlon | 21 (2.3) |
| Gymnastics | 20 (2.2) |
| Volleyball | 17 (1.9) |
| Swimming | 12 (1.3) |
| Cycling | 10 (1.1) |
| Ski | 8 (0.9) |
| Horse riding | 6 (0.7) |
| Fencing | 5 (0.5) |
| Climbing | 4 (0.4) |
| Figure skating | 4 (0.4) |
| Cheerleading | 2 (0.2) |
| Field hockey | 2 (0.2) |
| Hiking | 2 (0.2) |
| Canoe-kayak | 1 (0.1) |
| CrossFit | 1 (0.1) |
| Curling | 1 (0.1) |
| Golf | 1 (0.1) |
| Karting | 1 (0.1) |
| Multisport | 1 (0.1) |
| Queimada | 1 (0.1) |
| Rowing | 1 (0.1) |
| Sailing | 1 (0.1) |
| Skateboarding | 1 (0.1) |
|  |  |

**Supplementary Material 2 - Impact of social media according the level of practice and number of hours spent on social media**

|  | **Level of practice** | | | | | **Number of hours spent of social media** | | | |
| --- | --- | --- | --- | --- | --- | --- | --- | --- | --- |
|  | ***Amateur***  *(N=237)* | ***Competitive***  (N=239) | ***High-level***  (N=303) | ***World class or Elite***  *(N=133)* | *p-value* | ***Less then 2 hours / day***  *(N=295)* | ***2-4 hours***  ***/ day***  *(N=420)* | ***More than 4 hours / day***  *(N=197)* | *p-value* |
| **Influence on health** |  |  |  |  | 0.46 |  |  |  | **0.0056*** |
| Not at all | 27 (11.4) | 30 (12.5) | 46 (15.2) | 22 (16.5) |  | 53 (18.0) | 58 (13.8) | 14 (7.1) |  |
| A little or moderately | 160 (67.5) | 168 (70.3) | 208 (68.7) | 83 (62.4) |  | 194 (65.8) | 289 (68.8) | 136 (69.0) |  |
| Significantly or extremely | 50 (21.1) | 41 (17.1) | 49 (16.2) | 28 (21.0) |  | 48 (16.3) | 73 (17.4) | 47 (23.9) |  |
|  |  |  |  |  | 0.45 |  |  |  | **0.0013*** |
| Negative | 23 (9.7) | 27 (11.3) | 42 (13.9) | 15 (11.3) |  | 38 (12.9) | 37 (8.8) | 32 (16.2) |  |
| Positive | 52 (21.9) | 55 (23.0) | 82 (27.1) | 31 (23.3) |  | 81 (27.5) | 110 (26.2) | 29 (14.7) |  |
| Positive and negative | 162 (68.3) | 157 (65.7) | 179 (59.1) | 87 (65.4) |  | 176 (59.7) | 273 (65.0) | 136 (69.0) |  |
|  |  |  |  |  |  |  |  |  |  |
| **Impact on stress and anxiety** | 75 (31.7) | 67 (28.0) | 60 (19.8) | 30 (22.6) | **0.010*** | 69 (23.4) | 102 (24.3) | 61 (31.0) | 0.13 |
|  |  |  |  |  |  |  |  |  |  |
| **Impact on self confidence** |  |  |  |  | **0.042*** |  |  |  | 0.18 |
| No impact | 109 (46.0) | 120 (50.2) | 149 (49.2) | 50 (37.6) |  | 154 (52.2) | 195 (46.4) | 79 (40.1) |  |
| Positive impact | 21 (8.9) | 36 (15.1) | 40 (13.2) | 25 (18.8) |  | 40 (13.6) | 55 (13.1) | 27 (13.7) |  |
| Negative impact | 50 (21.1) | 30 (12.5) | 44 (14.5) | 22 (26.5) |  | 40 (13.6) | 66 (15.7) | 40 (20.3) |  |
| Don’t know | 57 (24.0) | 53 (22.2) | 70 (23.1) | 36 (27.1) |  | 61 (20.7) | 104 (24.8) | 51 (25.9) |  |
|  |  |  |  |  |  |  |  |  |  |
| **Impact on quality of sleep** |  |  |  |  | 0.93 |  |  |  | **<0.0001*** |
| No impact | 85 (35.9) | 88 (36.8) | 115 (38.0) | 43 (32.3) |  | 139 (47.1) | 153 (36.4) | 39 (19.8) |  |
| Positive impact | 4 (1.7) | 8 (3.3) | 9 (3.0) | 4 (3.0) |  | 10 (3.4) | 10 (2.4) | 5 (2.5) |  |
| Negative impact | 115 (48.5) | 113 (47.3) | 137 (45.2) | 70 (52.6) |  | 110 (37.3) | 199 (47.4) | 126 (64.0) |  |
| Don’t know | 33 (13.9) | 30 (12.5) | 42 (13.9) | 16 (12.0) |  | 36 (12.2) | 58 (13.8) | 27 (13.7) |  |
|  |  |  |  |  |  |  |  |  |  |
| **Impact on mood** |  |  |  |  | **0.038*** |  |  |  | **0.036*** |
| No impact | 121 (51.0) | 136 (56.9) | 165 (54.5) | 67 (50.4) |  | 172 (58.3) | 225 (53.6) | 92 (46.7) |  |
| Positive impact | 28 (11.8) | 28 (11.7) | 50 (16.5) | 15 (11.3) |  | 33 11.2) | 51 (12.1) | 37 (18.8) |  |
| Negative impact | 48 (20.2) | 42 (17.6) | 31 (10.2) | 22 (16.5) |  | 41 (13.9) | 63 (15.0) | 39 (19.8) |  |
| Don’t know | 40 (16.9) | 33 (13.8) | 57 (18.8) | 29 (21.8) |  | 49 (16.6) | 81 (19.3) | 29 (14.7) |  |
|  |  |  |  |  |  |  |  |  |  |
| **Impact on desire train** |  |  |  |  | 0.076 |  |  |  | **0.014*** |
| No impact | 100 (42.2) | 108 (45.2) | 149 (49.2) | 62 (46.6) |  | 150 (50.9) | 182 (43.3) | 87 (44.2) |  |
| Positive impact | 107 (45.1) | 105 (43.9) | 120 (39.5) | 44 (33.1) |  | 114 (38.6) | 189 (45.0) | 73 (37.1) |  |
| Negative impact | 11 (4.6) | 12 (5.0) | 18 (5.9) | 9 (6.8) |  | 11 (3.7) | 26 (6.2) | 13 (6.6) |  |
| Don’t know | 19 (8.0) | 14 (5.9) | 16 (5.3) | 18 (13.5) |  | 20 (6.8) | 23 (5.5) | 24 (12.2) |  |
|  |  |  |  |  |  |  |  |  |  |
| **Impact on performance** |  |  |  |  | 0.51 |  |  |  | 0.074 |
| No impact | 119 (50.2) | 136 (56.9) | 169 (55.8) | 73 (54.9) |  | 178 (60.3) | 213 (50.7) | 106 (53.8) |  |
| Positive impact | 59 (24.9) | 57 (23.9) | 61 (20.1) | 24 (18.0) |  | 58 (19.7) | 98 (23.3) | 45 (22.8) |  |
| Negative impact | 14 (5.9) | 9 (3.8) | 19 (6.3) | 6 (4.5) |  | 7 (2.4) | 29 (6.9) | 12 (6.1) |  |
| Don’t know | 45 (19.0) | 37 (15.5) | 54 (17.8) | 30 (22.6) |  | 52 (17.6) | 80 (19.0) | 34 (17.3) |  |
|  |  |  |  |  |  |  |  |  |  |
| **Impact on self-image** |  |  |  |  | 0.52 |  |  |  | **0.016*** |
| No impact | 124 (52.3) | 138 (57.7) | 155 (51.2) | 64 (48.1) |  | 172 (58.3) | 216 (51.4) | 93 (47.2) |  |
| Positive impact | 24 (10.1) | 31 (13.0) | 35 (11.5) | 16 (12.0) |  | 30 (10.2) | 56 (13.3) | 20 (10.1) |  |
| Negative impact | 40 (16.9) | 36 (15.1) | 56 (18.5) | 22 (16.5) |  | 38 (12.9) | 67 (16.0) | 49 (24.9) |  |
| Don’t know | 49 (20.7) | 34 (14.2) | 57 (18.8) | 31 (23.3) |  | 55 (18.6) | 81 (19.3) | 35 (17.8) |  |
|  |  |  |  |  |  |  |  |  |  |
| **Impact on eating habits** |  |  |  |  | 0.98 |  |  |  | 0.10 |
| No impact | 145 (61.2) | 135 (56.5) | 179 (59.1) | 80 (60.1) |  | 181 (61.4) | 254 (60.5) | 104 (52.8) |  |
| Positive impact | 46 (19.4) | 55 (23.0) | 63 (20.8) | 25 (18.8) |  | 52 (17.6) | 95 (22.6) | 42 (21.3) |  |
| Negative impact | 22 (9.3) | 28 (11.7) | 33 (10.9) | 14 (10.5) |  | 31 (10.5) | 36 (8.6) | 30 (15.2) |  |
| Don’t know | 24 (10.1) | 21 (8.8) | 28 (9.2) | 14 (10.5) |  | 31 (10.5) | 35 (8.3) | 21 (10.7) |  |
|  |  |  |  |  |  |  |  |  |  |
| **Affect motivation and athletic performance** |  |  |  |  | 0.64 |  |  |  | **0.0065*** |
| No | 112 (47.3) | 107 (44.8) | 153 (50.5) | 56 (42.1) |  | 163 (55.2) | 174 (41.4) | 91 (46.2) |  |
| Yes, positively | 76 (32.1) | 85 (35.6) | 91 (30.0) | 46 (34.6) |  | 89 (30.2) | 150 (35.7) | 59 (30.0) |  |
| Yes, negatively | 7 (3.0) | 6 (2.5) | 3 (1.0) | 2 (1.5) |  | 5 (1.7) | 7 (1.7) | 6 (3.0) |  |
| Yes, positively and negatively | 41 (17.3) | 38 (15.9) | 55 (18.1) | 26 (19.5) |  | 36 (12.2) | 83 (19.8) | 41 (20.8) |  |
| Other or don’t know | 1 (0.4) | 3 (1.3) | 1 (0.3) | 3 (2.3) |  | 2 (0.7) | 6 (1.4) | 0 (0.0) |  |
| **Pressure to maintain image** | 81 (34.2) | 69 (28.9) | 73 (24.1) | 43 (32.3) | 0.063 | 62 (21.0) | 123 (29.3) | 81 (41.1) | **<0.0001*** |
|  |  |  |  |  |  |  |  |  |  |
| **Relinquish image rights sponsors** | 56 (23.6) | 38 (15.9) | 62 (20.5) | 43 (32.3) | **0.0025*** | 49 (16.6) | 102 (24.3) | 48 (24.4) | **0.031*** |
|  |  |  |  |  |  |  |  |  |  |
| **Feeling of isolation or connection** |  |  |  |  | 0.067 |  |  |  | **0.0097*** |
| No effect | 146 (61.6) | 148 (61.9) | 180 (59.4) | 62 (46.6) |  | 169 (57.3) | 263 (62.6) | 104 (52.8) |  |
| More connected | 71 (30.0) | 68 (28.4) | 100 (33.0) | 55 (41.3) |  | 105 (35.6) | 123 (29.3) | 66 (33.5) |  |
| More isolated | 20 (8.4) | 20 (8.4) | 17 (5.6) | 13 (9.8) |  | 16 (5.4) | 29 (6.9) | 25 (12.7) |  |
| Both | 0 (0.0) | 3 (1.3) | 3 (1.0) | 2 (1.5) |  | 3 (1.0) | 3 (0.7) | 2 (1.0) |  |
| Other or don’t know | 0 (0.0) | 0 (0.0) | 3 (1.0) | 1 (0.8) |  | 2 (0.7) | 2 (0.5) | 0 (0.0) |  |
|  |  |  |  |  |  |  |  |  |  |
| **Impact on ability to focus on training and competitions** |  |  |  |  | 0.72 |  |  |  | 0.37 |
| (Very or rather) negative | 19 (8.0) | 20 (8.4) | 35 (11.6) | 16 (12.0) |  | 30 (10.2) | 34 (8.1) | 26 (13.2) |  |
| Neutral | 177 (74.7) | 176 (73.6) | 211 (69.6) | 93 (69.9) |  | 213 (72.2) | 310 (73.8) | 134 (68.0) |  |
| (Very or rather) positive | 41 (17.3) | 43 (18.0) | 57 (18.8) | 24 (18.0) |  | 52 (17.6) | 76 (18.1) | 37 (18.8) |  |
|  |  |  |  |  |  |  |  |  |  |
| **Received support or encouragement via social media** | 109 (46.0) | 140 (58.6) | 178 (58.8) | 94 (70.7) | **<0.0001*** | 143 (48.5) | 261 (62.1) | 117 (59.4) | **0.0010*** |

*Results are expressed as N(%) and p-value Chi-square test*

**Supplementary Material 3 - Factors influencing social media impact**

| **Factors** | **Explanatory variables** | **Simple logistic regression models** | | **Multiple logistic regression models** | |
| --- | --- | --- | --- | --- | --- |
|  |  | **OR (95%CI)** | **p-value** | **Adjusted OR (95%CI)** | **Adjusted p-value** |
| Pressure to maintain image | Sex, female (ref.=male) | 1.7 (1.3 – 2.3) | **0.0002*** | **1.7 (1.2 – 2.2)** | **0.0013*** |
|  | Age (ref= <18 years) | 1.9 (1.4 – 2.6) | **<0.0001*** | **1.6 (1.2 – 2.3)** | **0.0037*** |
|  |  |  |  |  |  |
|  | Snapchat use | 0.82 (0.54 – 1.2) | 0.33 | 0.90 (0.59 – 1.4) | 0.62 |
|  | Instagram use | 1.9 (1.2 – 3.1) | **0.0079*** | **1.7 (1.05 – 2.9)** | **0.030*** |
|  | Tik Tok use | 1.2 (0.85 – 1.7) | 0.29 | 1.2 (0.83 – 1.7) | 0.35 |
|  | Facebook use | 1.4 (0.87 – 2.2) | 0.17 | 1.0 (0.62 – 1.7) | 0.94 |
|  | Twitter use | 1.0 (0.56 – 2.0) | 0.88 | 1.0 (0.53 – 2.0) | 0.92 |
|  | Other social media use | 0.74 (0.45 – 1.2) | 0.25 | 0.85 (0.50 – 1.4) | 0.55 |
|  |  |  |  |  |  |
|  | Relinquish image rights sponsors | 2.3 (1.7 – 3.2) | **<0.0001*** | **2.3 (1.7 – 3.3)** | **<0.0001*** |
|  | Team sport (ref= individual) | 1.0 (0.74 – 1.3) | 0.99 | - | - |
|  | Sports betting | 0.77 (0.54 – 1.1) | 0.16 | - | - |
|  | Level of practice (ref=amateur) | 1.4 (1.001 – 1.9) | **0.049*** | 1.2 (0.90 – 1.8) | 0.19 |
| Impact on stress and anxiety | Sex, female (ref.=male) | 1.7 (1.3 – 2.3) | **0.0004*** | **1.7 (1.2 – 2.3)** | **0.0012*** |
|  | Age (ref= <18 years) | 1.8 (1.3 – 2.4) | **0.0002*** | 1.4 (0.995 – 1.7) | 0.053 |
|  |  |  |  |  |  |
|  | Snapchat use | 0.68 (0.45 – 1.03) | 0.072 | 0.77 (0.50 – 1.2) | 0.22 |
|  | Instagram use | 1.8 (1.1 – 2.9) | **0.019*** | 1.6 (0.98 – 2.7) | 0.061 |
|  | Tik Tok use | 0.87 (0.61 – 1.2) | 0.44 | 0.85 (0.59 – 1.2) | 0.38 |
|  | Facebook use | 1.5 (0.94 – 2.4) | 0.087 | 1.2 (0.75 – 2.0) | 0.41 |
|  | Twitter use | 1.7 (0.92 – 3.2) | 0.091 | 1.7 (0.88 – 3.1) | 0.12 |
|  | Other social media use | 0.93 (0.57 – 1.5) | 0.78 | 0.98 (0.59 – 1.6) | 0.93 |
|  |  |  |  |  |  |
|  | Relinquish image rights sponsors | 1.3 (0.90 – 1.8) | 0.18 | - | - |
|  | Team sport (ref= individual) | 0.92 (0.68 – 1.2) | 0.62 | - | - |
|  | Sports betting | 0.85 (0.59 – 1.2) | 0.39 | - | - |
|  | Level of practice (ref=amateur) | 1.5 (1.1 – 2.1) | **0.011*** | 0.4 (0.97 – 1.9) | 0.073 |
| Impact on self confidence | Sex, female (ref.=male) | 2.2 (1.6 – 3.0) | **<0.0001*** | **2.2 (1.5 – 3.0)** | **<0.0001*** |
|  | Age (ref= <18 years) | 1.4 (1.04 – 2.0) | **0.026*** | 1.0 (0.71 – 1.5) | 0.89 |
|  |  |  |  |  |  |
|  | Snapchat use | 0.56 (0.36 – 0.85) | **0.0068*** | **0.58 (0.37 – 0.91)** | **0.017*** |
|  | Instagram use | 1.9 (1.2 – 3.1) | **0.0091*** | **1.8 (1.1 – 3.1)** | **0.023*** |
|  | Tik Tok use | 1.3 (0.88 – 1.9) | 0.20 | 1.3 (0.86 – 1.9) | 0.22 |
|  | Facebook use | 1.2 (0.71 – 1.9) | 0.56 | 1.0 0.60 – 1.7) | 0.99 |
|  | Twitter use | 1.8 (0.95 – 3.5) | 0.068 | **2.3 (1.1 – 4.6)** | **0.019*** |
|  | Other social media use | 0.92 (0.55 – 1.5) | 0.75 | 1.0 (0.61 – 1.8) | 0.87 |
|  |  |  |  |  |  |
|  | Relinquish image rights sponsors | 2.0 (1.4 – 2.9) | **0.0002*** | **2.2 (1.5 – 3.3)** | **<0.0001*** |
|  | Team sport (ref= individual) | 0.60 (0.44 – 0.82) | **0.0014*** | **0.65 (0.46 – 0.92)** | **0.014*** |
|  | Sports betting | 1.1 (0.77 – 1.6) | 0.59 | - | - |
|  | Level of practice (ref=amateur) | 1.0 (0.74 – 1.5) | 0.76 |  |  |
| Impact on quality of sleep | Sex, female (ref.=male) | 1.1 (0.87 – 1.5) | 0.32 | - | - |
|  | Age (ref= <18 years) | 2.0 (1.5 – 2.8) | **<0.0001*** | **1.8 (1.3 – 2.6)** | **0.0004*** |
|  |  |  |  |  |  |
|  | Snapchat use | 0.94 0.63 – 1.4) | 0.76 | 1.0 (0.70 – 1.6) | 0.83 |
|  | Instagram use | 1.6 (1.0 – 2.4) | **0.030*** | 1.4 (0.90 – 2.1) | 0.14 |
|  | Tik Tok use | 1.4 (1.0 – 2.0) | **0.050*** | **1.5 (1.1 – 2.2)** | **0.017*** |
|  | Facebook use | 1.4 (0.81 – 2.3) | 0.23 | 1.1 (0.66 – 2.0) | 0.65 |
|  | Twitter use | 3.0 (1.3 – 6.7) | **0.0068*** | **2.6 (1.1 – 5.7)** | **0.023*** |
|  | Other social media use | 0.91 (0.59 – 1.4) | 0.69 | 1.0 (0.65 – 1.6) | 0.94 |
|  |  |  |  |  |  |
|  | Relinquish image rights sponsors | 1.1 (0.79 – 1.6) | 0.55 | - | - |
|  | Team sport (ref= individual) | 1.1 (0.84 – 1.5) | 0.43 | - | - |
|  | Sports betting | 1.0 (0.74 – 1.5) | 0.80 | - | - |
|  | Level of practice (ref=amateur) | 1.0 (0.73 – 1.4) | 0.95 |  |  |
| Impact on mood | Sex, female (ref.=male) | 1.1 (0.83 – 1.5) | 0.44 | - | - |
|  | Age (ref= <18 years) | 0.95 (0.70 – 1.3) | 0.77 | - | - |
|  |  |  |  |  |  |
|  | Snapchat use | 0.53 (0.35 – 0.80) | **0.0027*** | 0.69 (0.46 – 1.03) | 0.067 |
|  | Instagram use | 1.3 (0.80 – 2.0) | 0.30 | **1.7 (1.1 – 2.8)** | **0.022*** |
|  | Tik Tok use | 1.3 (0.90 – 1.9) | 0.15 | 1.3 (0.91 – 1.8) | 0.15 |
|  | Facebook use | 1.1 (0.65 – 1.8) | 0.75 | **1.7 (1.1 – 2.7)** | **0.024*** |
|  | Twitter use | 1.9 (1.003 – 3.6) | **0.049*** | 1.5 (0.81 – 2.8) | 0.20 |
|  | Other social media use | 1.2 (0.76 – 2.0) | 0.39 | 0.98 (0.61 – 1.6) | 0.95 |
|  |  |  |  |  |  |
|  | Relinquish image rights sponsors | 0.90 (0.62 – 1.3) | 0.56 | - | - |
|  | Team sport (ref= individual) | 1.0 (0.74 – 1.4) | 0.94 | - | - |
|  | Sports betting | 1.6 (1.1 – 2.3) | **0.0070*** | 1.2 (0.82 – 1.7) | 0.38 |
|  | Level of practice (ref=amateur) | 1.2 (0.88 – 1.7) | 0.23 |  |  |
| Impact on desire to train | Sex, female (ref.=male) | 0.92 (0.70 – 1.2) | 0.57 | - | - |
|  | Age (ref= <18 years) | 1.5 (1.1 – 1.9) | **0.0097*** | 1.3 (0.98 – 1.9) | 0.070 |
|  |  |  |  |  |  |
|  | Snapchat use | 0.74 (0.50 – 1.1) | 0.12 | 0.77 (0.52 – 1.1) | 0.20 |
|  | Instagram use | 1.8 (1.2 – 2.7) | **0.0079*** | **1.7 (1.1 – 2.6)** | **0.024*** |
|  | Tik Tok use | 1.0 (0.74 – 1.4) | 0.90 | 1.1 (0.78 – 1.5) | 0.59 |
|  | Facebook use | 0.79 (0.50 – 1.3) | 0.32 | 0.71 (0.44 – 1.1) | 0.17 |
|  | Twitter use | 1.9 (0.97 – 3.6) | 0.063 | 1.8 (0.91 – 3.5) | 0.092 |
|  | Other social media use | 0.67 (0.43 – 1.04) | 0.077 | 0.73 (0.47 – 1.1) | 0.17 |
|  |  |  |  |  |  |
|  | Relinquish image rights sponsors | 1.5 (1.1 – 2.1) | **0.018*** | **1.5 (1.1 – 2.1)** | **0.020*** |
|  | Team sport (ref= individual) | 0.78 (0.59 – 1.02) | 0.073 | 0.79 (0.59 – 1.05) | 0.10 |
|  | Sports betting | 1.4 (1.0 – 2.0) | **0.032*** | **1.5 (1.1 – 2.1)** | **0.019*** |
|  | Level of practice (ref=amateur) | 1.2 (0.90 – 1.7) | 0.20 |  |  |
| Impact on performance | Sex, female (ref.=male) | 0.99 (0.73 – 1.4) | 0.97 | - | - |
|  | Age (ref= <18 years) | 1.1 (0.81 – 1.5) | 0.50 | - | - |
|  |  |  |  |  |  |
|  | Snapchat use | 0.90 (0.58 – 1.4) | 0.61 | - | - |
|  | Instagram use | 1.2 (0.78 – 2.0) | 0.36 | - | - |
|  | Tik Tok use | 1.2 (0.80 – 1.7) | 0.43 | - | - |
|  | Facebook use | 1.4 (0.90 – 2.3) | 0.12 | - | - |
|  | Twitter use | 1.6 (0.83 – 3.0) | 0.17 | - | - |
|  | Other social media use | 1.0 (0.63 – 1.7) | 0.87 | - | - |
|  |  |  |  |  |  |
|  | Relinquish image rights sponsors | 1.6 (1.1 – 2.3) | **0.0090*** | **1.6 (1.1 – 2.3)** | **0.0090*** |
|  | Team sport (ref= individual) | 0.84 (0.62 – 1.2) | 0.29 | - | - |
|  | Sports betting | 1.1 (0.80 – 1.7) | 0.45 | - | - |
|  | Level of practice (ref=amateur) | 1.3 (0.94 – 1.9) | 0.11 |  |  |
| Impact on self-image | Sex, female (ref.=male) | 2.3 (1.7 – 3.2) | **<0.0001*** | **2.3 (1.7 – 3.1)** | **<0.0001*** |
|  | Age (ref= <18 years) | 1.3 (0.99 – 1.9) | 0.058 | 1.1 (0.81 – 1.6) | 0.45 |
|  |  |  |  |  |  |
|  | Snapchat use | 0.72 (0.48 – 1.1) | 0.13 | 0.79 (0.51 – 1.2) | 0.28 |
|  | Instagram use | 1.7 (1.03 – 2.7) | **0.037*** | 1.6 (0.96 – 2.7) | 0.071 |
|  | Tik Tok use | 1.3 (0.89 – 1.8) | 0.19 | 1.2 (0.79 – 1.7) | 0.45 |
|  | Facebook use | 0.95 (0.58 – 1.6) | 0.84 | 0.82 (0.48 – 1.4) | 0.45 |
|  | Twitter use | 1.0 (0.50 – 2.0) | 0.98 | 1.1 (0.54 – 2.3) | 0.77 |
|  | Other social media use | 0.71 (0.42 – 1.2) | 0.20 | 0.77 (0.45 – 1.3) | 0.34 |
|  |  |  |  |  |  |
|  | Relinquish image rights sponsors | 1.7 (1.2 – 2.4) | **0.0040*** | **1.7 (1.2 – 2.6)** | **0.0025*** |
|  | Team sport (ref= individual) | 0.80 (0.59 – 1.1) | 0.15 | - | - |
|  | Sports betting | 1.1 (0.80 – 1.7) | 0.45 | - | - |
|  | Level of practice (ref=amateur) | 0.94 (0.66 – 1.3) | 0.73 |  |  |
| Impact on eating habits | Sex, female (ref.=male) | 1.7 (1.2 – 2.2) | **0.0006*** | **1.6 (1.2 – 2.1)** | **0.0027*** |
|  | Age (ref= <18 years) | 1.6 (1.1 – 2.1) | **0.0037*** | 1.2 (0.89 – 1.7) | 0.21 |
|  |  |  |  |  |  |
|  | Snapchat use | 0.69 (0.46 – 1.04) | 0.074 | 0.71 (0.47 – 1.1) | 0.11 |
|  | Instagram use | 1.7 (1.1 – 2.7) | **0.024*** | **1.6 (1.0 – 2.6)** | **0.050*** |
|  | Tik Tok use | 1.3 (0.91 – 1.8) | 0.15 | 1.2 (0.86 – 1.8) | 0.25 |
|  | Facebook use | 1.7 (1.1 – 2.7) | **0.026*** | 1.5 (0.94 – 2.5) | 0.087 |
|  | Twitter use | 1.5 (0.82 – 2.8) | 0.19 | 1.6 (0.84 – 3.0) | 0.15 |
|  | Other social media use | 0.99 (0.62 – 1.6) | 0.98 | 1.0 (0.63 – 1.7) | 0.94 |
|  |  |  |  |  |  |
|  | Relinquish image rights sponsors | 1.1 (0.80 – 1.6) | 0.49 | - | - |
|  | Team sport (ref= individual) | 0.85 (0.63 – 1.1) | 0.27 | - | - |
|  | Sports betting | 1.1 (0.79 – 1.6) | 0.51 | - | - |
|  | Level of practice (ref=amateur) | 0.85 (0.61 – 1.2) | 0.33 |  |  |
| Impact on motivation | Sex, female (ref.=male) | 1.1 (0.85 – 1.4) | 0.45 | - | - |
|  | Age (ref= <18 years) | 1.4 (1.1 – 1.9) | **0.012*** | 1.2 (0.92 – 1.7) | 0.15 |
|  |  |  |  |  |  |
|  | Snapchat use | 0.94 (0.65 – 1.4) | 0.73 | 1.0 (0.71 – 1.5) | 0.87 |
|  | Instagram use | 1.8 (1.2 – 2.8) | **0.0028*** | **1.7 (1.1 – 2.5)** | **0.012*** |
|  | Tik Tok use | 0.95 (0.69 – 1.3) | 0.76 | 1.1 (0.77 – 1.5) | 0.73 |
|  | Facebook use | 1.3 (0.82 – 2.0) | 0.26 | 1.2 (0.73 – 1.9) | 0.51 |
|  | Twitter use | 1.2 (0.65 – 2.2) | 0.58 | 1.1 (0.61 – 2.1) | 0.68 |
|  | Other social media use | 0.86 (0.57 – 1.3) | 0.49 | 0.92 (0.60 – 1.4) | 0.72 |
|  |  |  |  |  |  |
|  | Relinquish image rights sponsors | 1.4 (1.0 – 1.9) | **0.050*** | 1.3 (0.96 – 1.8) | 0.086 |
|  | Team sport (ref= individual) | 0.65 (0.50 – 0.85) | **0.019*** | **0.62 (0.47 – 0.82)** | **0.0009*** |
|  | Sports betting | 1.1 (0.78 – 1.5) | 0.64 | - | - |
|  | Level of practice (ref=amateur) | 0.99 (0.74 – 1.3) | 0.97 |  |  |
| Feeling of isolation | Sex, female (ref.=male) | 0.76 (0.47 – 1.2) | 0.26 | - | - |
|  | Age (ref= <18 years) | 1.4 (0.86 – 2.2) | 0.18 | - | - |
|  |  |  |  |  |  |
|  | Snapchat use | 0.83 (0.44 – 1.6) | 0.56 | - | - |
|  | Instagram use | 1.3 (0.61 – 2.5) | 0.54 | - | - |
|  | Tik Tok use | 0.78 (0.45 – 1.3) | 0.36 | - | - |
|  | Facebook use | 1.2 (0.60 – 2.6) | 0.57 | - | - |
|  | Twitter use | 1.4 (0.56 – 3.5) | 0.47 | - | - |
|  | Other social media use | 0.85 (0.39 – 1.8) | 0.67 | - | - |
|  |  |  |  |  |  |
|  | Relinquish image rights sponsors | 2.3 (1.4 – 3.8) | **0.0009*** | **2.3 (1.4 – 3.8)** | **0.0009*** |
|  | Team sport (ref= individual) | 1.1 (0.66 – 1.7) | 0.78 | - | - |
|  | Sports betting | 1.1 (0.65 – 2.0) | 0.68 | - | - |
|  | Level of practice (ref=amateur) | 0.97 (0.57 – 1.7) | 0.92 |  |  |

**Supplementary Material 4 - Impact of online abuse** (N=95 victims)

|  | All (N=95) | Related to sports (N=29) | Unrelated to sports (N=66) |
| --- | --- | --- | --- |
| **Impact on self confidence** |  |  |  |
| No impact | 25 (26.3) | 9 (31.0) | 16 (24.2) |
| Low impact | 18 (18.9) | 6 (20.7) | 12 (18.2) |
| Moderate impact | 26 (27.4) | 5 (17.2) | 21 (31.8) |
| Severe impact | 17 (17.9) | 5 (17.2) | 12 (18.2) |
| Very severe impact | 7 (7.4) | 2 (6.9) | 5 (7.6) |
| Don’t know | 2 (2.1) | 2 (6.9) | 0 (0.0) |
|  |  |  |  |
| **Impact on sleep quality** |  |  |  |
| No impact | 33 (34.7) | 15 (51.7) | 18 (27.3) |
| Low impact | 16 (16.8) | 3 (10.3) | 13 (19.7) |
| Moderate impact | 22 (23.2) | 2 (6.9) | 20 (30.3) |
| Severe impact | 13 (13.7) | 6 (20.7) | 7 (10.6) |
| Very severe impact | 7 (7.4) | 1 (3.4) | 6 (9.1) |
| Don’t know | 4 (4.2) | 2 (6.9) | 2 (3.0) |
|  |  |  |  |
| **Impact on mood** |  |  |  |
| No impact | 17 (17.9) | 7 (24.1) | 10 (15.2) |
| Low impact | 16 (16.8) | 5 (17.2) | 11 (16.7) |
| Moderate impact | 20 (21.1) | 5 (17.2) | 15 (22.7) |
| Severe impact | 27 (28.4) | 9 (31.0) | 18 (27.3) |
| Very severe impact | 9 (9.5) | 2 (6.9) | 7 (10.6) |
| Don’t know | 6 (6.3) | 1 (3.4) | 5 (7.6) |
|  |  |  |  |
| **Impact on desire to train** |  |  |  |
| No impact | 46 (48.4) | 13 (44.8) | 33 (50.0) |
| Low impact | 7 (7.4) | 3 (10.3) | 4 (6.1) |
| Moderate impact | 14 (14.7) | 2 (6.9) | 12 (18.2) |
| Severe impact | 13 (13.7) | 6 (20.7) | 7 (10.6) |
| Very severe impact | 9 (9.5) | 4 (13.8) | 5 (7.6) |
| Don’t know | 6 (6.3) | 1 (3.4) | 5 (7.6) |
|  |  |  |  |
| **Impact on performance** |  |  |  |
| No impact | 43 (45.3) | 13 (44.8) | 30 (45.5) |
| Low impact | 11 (11.6) | 3 (10.3) | 8 (12.1) |
| Moderate impact | 17 (17.9) | 6 (20.7) | 11 (16.7) |
| Severe impact | 10 (10.5) | 1 (3.4) | 9 (13.6) |
| Very severe impact | 7 (7.4) | 3 (10.3) | 4 (6.1) |
| Don’t know | 7 (7.4) | 3 (10.3) | 4 (6.1) |
|  |  |  |  |
| **Impact on self-image** |  |  |  |
| No impact | 24 (25.3) | 10 (34.5) | 14 (21.2) |
| Low impact | 13 (13.7) | 4 (13.8) | 9 (13.6) |
| Moderate impact | 20 (21.1) | 6 (20.7) | 14 (21.2) |
| Severe impact | 19 (20.0) | 6 (20.7) | 13 (19.7) |
| Very severe impact | 15 (15.8) | 1 (3.4) | 14 (21.2) |
| Don’t know | 4 (4.2) | 2 (6.9) | 2 (3.0) |
|  |  |  |  |
| **Impact on eating habits** |  |  |  |
| No impact | 48 (50.5) | 20 (69.0) | 28 (42.4) |
| Low impact | 13 (13.7) | 2 (6.9) | 11 (16.7) |
| Moderate impact | 14 (14.7) | 2 (6.9) | 12 (18.2) |
| Severe impact | 9 (9.5) | 1 (3.4) | 8 (12.1) |
| Very severe impact | 7 (7.4) | 2 (6.9) | 5 (7.6) |
| Don’t know | 4 (4.2) | 2 (6.9) | 2 (3.0) |
| **Looking for help** | **34 (35.8)** | **8 (27.6)** | **26 (39.4)** |
| To famility/friends | 22 (64.7) | 4 (50.0) | 18 (69.2) |
| To health prof. | 7 (20.6) | 2 (25.0) | 5 (19.2) |
| To coach | 2 (5.9) | 1 (12.5) | 1 (3.8) |
| To others | 3 (8.8) | 1 (12.5) | 2 (7.7) |
